# Plasma Biomarker of Dietary Phytosterol Intake

**DOI:** 10.1371/journal.pone.0116912

**Published:** 2015-02-10

**Authors:** Xiaobo Lin, Susan B. Racette, Lina Ma, Michael Wallendorf, Catherine Anderson Spearie, Richard E. Ostlund

**Affiliations:** 1 Division of Endocrinology, Metabolism & Lipid Research, Department of Medicine, Institute for Clinical and Translational Sciences, Washington University School of Medicine, 660 South Euclid Ave., St. Louis, MO, 63110, United States of America; 2 Program in Physical Therapy, Institute for Clinical and Translational Sciences, Washington University School of Medicine, 660 South Euclid Ave., St. Louis, MO, 63110, United States of America; 3 Division of Biostatistics, Institute for Clinical and Translational Sciences, Washington University School of Medicine, 660 South Euclid Ave., St. Louis, MO, 63110, United States of America; 4 Lifestyle Intervention Research Core, Institute for Clinical and Translational Sciences, Washington University School of Medicine, 660 South Euclid Ave., St. Louis, MO, 63110, United States of America; Oklahoma State University, UNITED STATES

## Abstract

**Background:**

Dietary phytosterols, plant sterols structurally similar to cholesterol, reduce intestinal cholesterol absorption and have many other potentially beneficial biological effects in humans. Due to limited information on phytosterol levels in foods, however, it is difficult to quantify habitual dietary phytosterol intake (DPI). Therefore, we sought to identify a plasma biomarker of DPI.

**Methods and Findings:**

Data were analyzed from two feeding studies with a total of 38 subjects during 94 dietary periods. DPI was carefully controlled at low, intermediate, and high levels. Plasma levels of phytosterols and cholesterol metabolites were assessed at the end of each diet period. Based on simple ordinary least squares regression analysis, the best biomarker for DPI was the ratio of plasma campesterol to the endogenous cholesterol metabolite 5-α-cholestanol (R^2^ = 0.785, *P* < 0.0001). Plasma campesterol and 5-α-cholestanol levels varied greatly among subjects at the same DPI level, but were positively correlated at each DPI level in both studies (r > 0.600; *P* < 0.01).

**Conclusion:**

The ratio of plasma campesterol to the coordinately regulated endogenous cholesterol metabolite 5-α-cholestanol is a biomarker of dietary phytosterol intake. Conversely, plasma phytosterol levels alone are not ideal biomarkers of DPI because they are confounded by large inter-individual variation in absorption and turnover of non-cholesterol sterols. Further work is needed to assess the relation between non-cholesterol sterol metabolism and associated cholesterol transport in the genesis of coronary heart disease.

## Introduction

In addition to their robust effects on cholesterol metabolism [1–3], phytosterols are reported to have many other biological actions, such as anti-inflammatory and anti-cancer effects [4,5]. However, it is difficult to quantify habitual dietary phytosterol intake (DPI) because phytosterol levels in foods are not systematically included in food databases. Even though food frequency questionnaire has been used to recall food intake of nutrients, it is more subjective in nature and more challenging, especially for older populations [6]. Therefore, a suitable plasma biomarker for DPI would be helpful in assessing the biological effects of dietary phytosterols in epidemiological studies.

Many diet-related investigations rely on measurement of plasma levels of the nutrient being studied (phytosterols in this case) in order to estimate habitual dietary intake. At first glance, that approach seems attractive with respect to phytosterols because they are not synthesized in humans. Therefore any phytosterols measured in plasma must have originated in the diet. However, phytosterols differ from other nutrients in a fundamental way; their plasma levels reflect a very complex relationship not only to dietary intake but also to several metabolic variables. Plasma phytosterol levels are affected by their intestinal absorption efficiency, mediated by intestinal lipid transporters Niemann-Pick C1-Like 1 (NPC1L1) (favoring sterol uptake) [7] and ATP-binding cassette G5 and G8 (ABCG5/ABCG8) transporters (favoring phytosterol efflux from enterocytes back to the lumen) [8,9]. Plasma phytosterols also are affected by hepatic NPC1L1 and ABCG5/ABCG8 transporters [10], which result in the rapid and near complete biliary excretion of phytosterols. Moreover, increasing phytosterol intake results in a plateau in plasma phytosterol levels, indicating competition of phytosterols for their own absorption [11,12]. Taken together, these results suggest that plasma phytosterol levels are dependent not only on DPI, but also on the absorption efficiency and the re-excretion rate of absorbed phytosterols. Therefore, plasma phytosterol levels alone are not ideal biomarkers for DPI.

**Fig 1:**
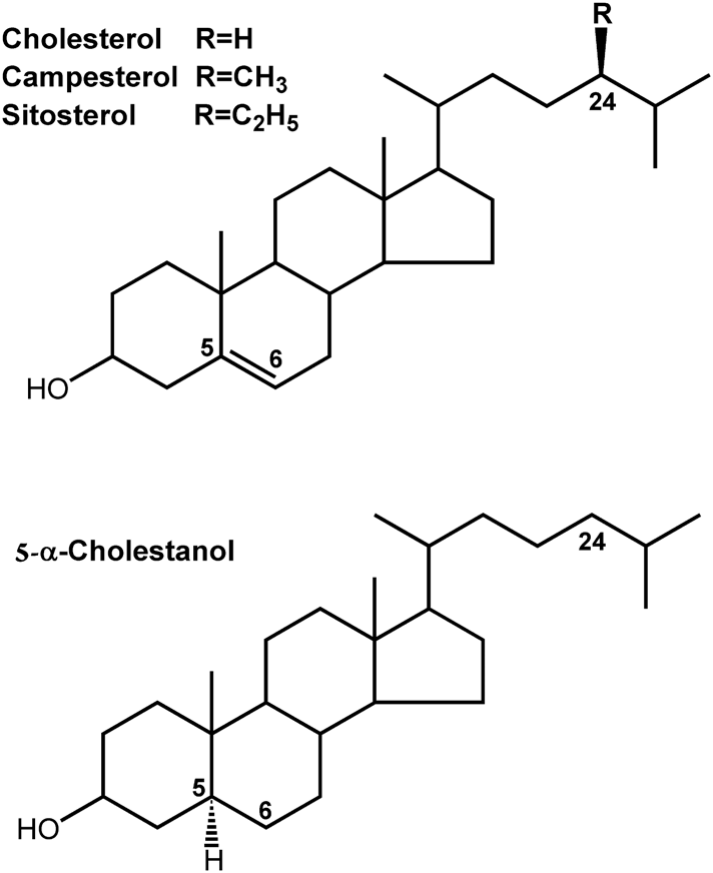
Sterol structures. Top panel shows the structures of cholesterol and the phytosterols campesterol and sitosterol. Bottom panel shows the structure of 5-α-cholestanol, the reduced form of cholesterol.

The endogenous cholesterol metabolite 5-α-cholestanol (5α-cholestan-3β-ol), an intermediate of bile acid synthesis, derives primarily from the catabolism of cholesterol in the liver [13,14]. Dietary intake of 5-α-cholestanol is very low and its contribution to plasma levels is thought to be negligible [15]. Phytosterols (e.g., campesterol, sitosterol) and 5-α-cholestanol are non-cholesterol sterols that are structurally similar to cholesterol. The major differences are saturation of the Δ5 double bond at the 5α position (5-α-cholestanol) and methyl and ethyl groups at position C-24 (campesterol and sitosterol, respectively) (Fig. 1). Average cholesterol absorption efficiency in humans is 56%, whereas phytosterols are not absorbed to a great extent, with absorption efficiency of campesterol being only 1.9% [16]. Campesterol is a common dietary phytosterol and has the highest intestinal absorption efficiency among individual phytosterols. In comparison, the intestinal absorption efficiency of the metabolite 5-α-cholestanol is 3.3% [17], resembling that of phytosterols rather than cholesterol.

The aim of this study was to identify a plasma biomarker for dietary phytosterol intake. We hypothesized that plasma phytosterol levels (total phytosterols or campesterol) would be suitable after normalization by a marker that reflects overall handling (i.e., absorption and excretion) of non-cholesterol sterols by the body.

## Methods

The current study is an analysis of results of two independent clinical studies in which we investigated the effects on cholesterol metabolism from supplemental phytosterols (Supplement Study, NCT00860054) [2] and phytosterols naturally present in the diet (Natural Study, NCT00860509) [3]. Participant characteristics, study designs, and outcome measures have been published [2,3]. Briefly, both studies were randomized, cross-over feeding trials in which all meals were prepared in a metabolic kitchen.

In the Supplement Study, 18 adults received a low-phytosterol diet (50 mg phytosterols/2000 kcal) plus beverages supplemented with 0, 400, or 2000 mg phytosterols/day for 4 weeks each, in random order. In the Natural Study, 20 subjects consumed two diets for 4 weeks each. The diets differed in phytosterol content from natural foods (126 mg phytosterols/2000 kcal vs. 449 mg phytosterols/2000 kcal), but otherwise were matched for nutrient content. In both studies, concentrations of plasma phytosterols, 5-α-cholestanol, and the cholesterol precursor lathosterol were determined by gas chromatography—mass spectrometry [2,3].

### Statistical Analysis

Statistical analysis was performed with SAS software (Version 9.3, SAS Institute Inc., Cary, NC) on data from the two feeding studies [2,3]. For each study, one-way ANOVA in the Mixed procedure was used to analyze treatment effects on the observed dietary phytosterol intake with Tukey adjustment for multiple comparisons. Spearman’s rank-order correlation was performed between plasma 5-α-cholestanol and campesterol levels at each DPI level, and between plasma 5-α-cholestanol and DPI in each study. Linear association of campesterol on 5-α-cholestanol was analyzed with mixed random effects repeated measures regression with a 5-α-cholestanol by treatment interaction term to test differences in slopes between treatments.

Combined data from the 2 studies were analyzed using repeated-measures regression models. R-Square statistics of simple ordinary least squares regression were used to compare outcome variables for strength of relationship to DPI with DPI as the independent variable. Dependent variables included individual phytosterols, total phytosterols, and their ratios to cholesterol and to 5-α-cholestanol. Repeated measures regression models were fit with the Mixed Procedure and included period nested within study as the repeated effect and a first order autoregressive correlation structure within subject. Data were log transformed when appropriate.

$$\text{Model}:\;LN(Y_{ij})=\beta_{0}+\beta_{1}[LN(DPI)]+e_{ij,}$$

where the dependent variable LN(Y_ij_) is the natural log of Y_ij_, which represents the ratio of plasma total phytosterol levels or campesterol levels to 5-α-cholestanol, β_0_ is the intercept, β_1_ is the slope, LN(DPI) is the natural log of DPI, and *e*_*ij*_ is the residual of subject i in period j.

## Results

**Fig 2:**
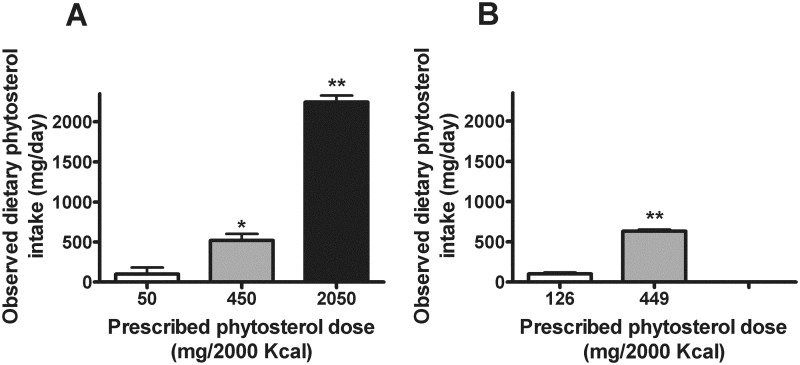
Observed dietary phytosterol intake (DPI) (A, Supplement Study; B, Natural Study). *significantly different from 50 mg/2000 Kcal, *P* < 0.01; **significantly different from 50 mg/2000 Kcal or 450 mg/2000 Kcal (A), or from 126 mg/2000 Kcal (B), *P* < 0.0001.

Subject characteristics at baseline are presented in Table 1. Based on the design of the controlled feeding studies and excellent adherence to the provided diets, the observed DPI differed between diet conditions. In the Supplement Study, DPI values at the three phytosterol doses (i.e., 50, 450, and 2050 mg/2000 Kcal) averaged 99 ± 82 (SEM), 520 ± 82, and 2244 ± 82 mg/day, respectively (Fig. 2A). In the Natural Study, DPI at the two phytosterol doses (i.e., 126 and 449 mg/2000 Kcal) averaged 101 ± 18 and 632 ± 18 mg/day (Fig. 2B).

**Table 1:** Participant characteristics at baseline.

Women/men	28/10
Age (y)	60.0 ± 12.4
Weight (kg)	75.4 ± 13.9
BMI (kg/m^2^)	27.0 ± 3.6
Lipids	
Total cholesterol (mg/dL)	223 ± 25
LDL cholesterol (mg/dL)	142 ± 19
HDL cholesterol (mg/dL)	61 ± 14
Triglyceride (mg/dL)	110 ± 36
Total cholesterol:HDL cholesterol	3.8 ± 0.7
Glucose (mg/dL)	95 ± 9
Blood pressure:	
Systolic (mm Hg)	118 ± 18
Diastolic (mm Hg)	74 ± 8

Values are means ± SD.

**Fig 3:**
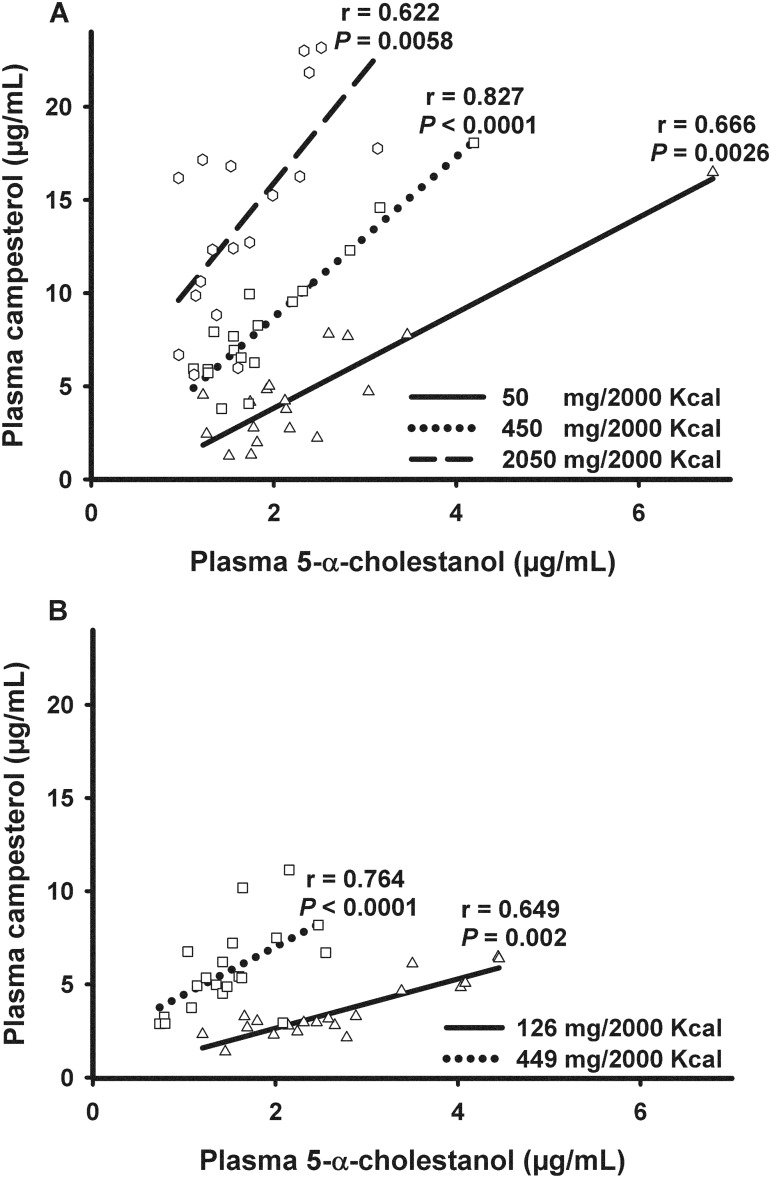
Correlations between plasma non-cholesterol sterol concentrations at each dietary phytosterol intake level. Values reflect Spearman’s rank-order correlation coefficients in the Supplement Study (A) and the Natural Study (B).

At each level of DPI, there was wide variability of plasma campesterol (1.26–16.49, 3.80–18.07, and 5.62–23.17 μg/mL at the three phytosterol doses in the Supplement Study; 1.38–6.48 and 2.88–11.14 μg/mL at the two phytosterol doses in the Natural Study) and 5-α-cholestanol concentrations (1.22–6.81, 1.12–4.19, 0.96–3.14 μg/mL, respectively, for Supplement Study; 1.20–4.45 and 0.73–2.55 μg/mL for Natural Study), suggesting that individual subjects handle non-cholesterol sterols differently (Fig. 3). In addition, plasma campesterol and 5-α-cholestanol were positively correlated at each of the three DPI levels in the Supplement Study (Fig. 3A), and at both DPI levels in the Natural Study (Fig. 3B). In the overall data, there also was a significant and positive association between plasma campesterol and plasma 5-α-cholestanol (*P* < 0.001) and the slope of this relation differed by phytosterol dose (*P* < 0.0001). On the other hand, there was a significant negative correlation between plasma 5-α-cholestanol and DPI in the Supplement Study (r = -0.4240, *P* = 0.0014) and in the Natural Study (r = -0.5947, *P* < 0.0001).

**Fig 4:**
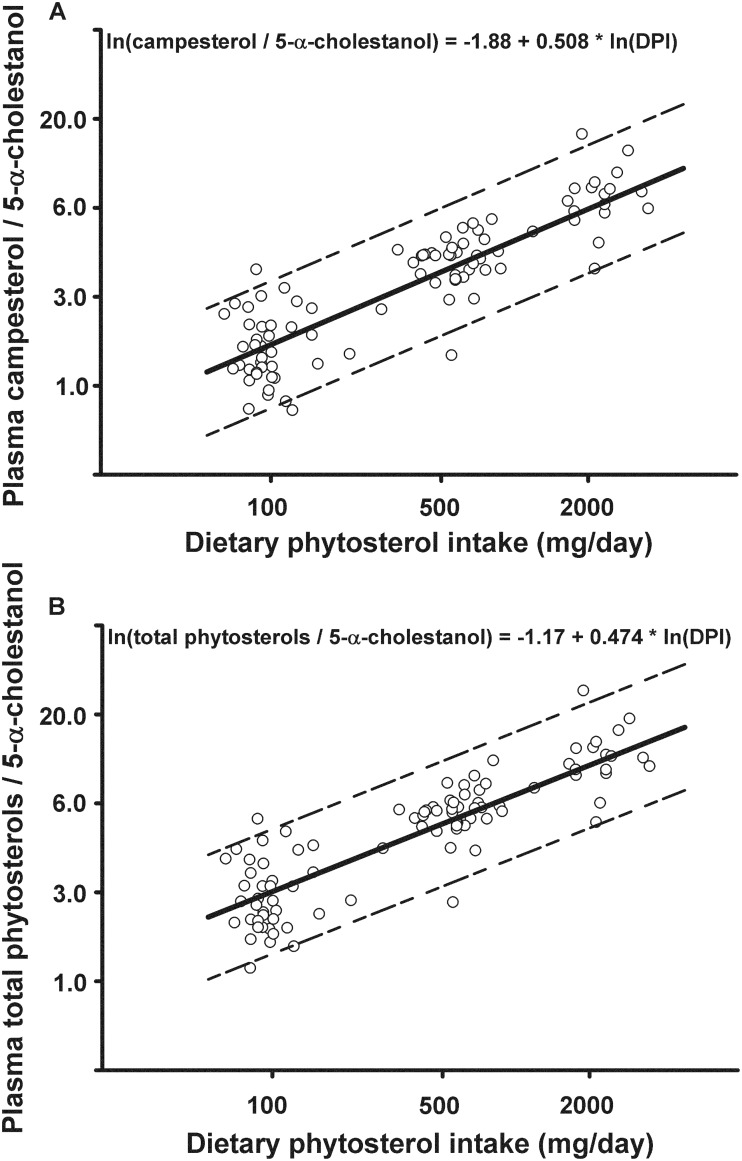
Regression of plasma phytosterol levels normalized by 5-α-cholestanol on dietary phytosterol intake (DPI). All values were transformed to natural log. Dotted lines represent 95% prediction limits of Ln(Campesterol/5-α-cholestanol) (A) and Ln(Total phytosterols/5-α-cholestanol) (B).

As shown in Table 2, the variable most strongly correlated with DPI was the ratio of plasma campesterol to 5-α-cholestanol (R^2^ = 0.785, *P* < 0.0001), followed by the ratio of plasma total phytosterols to 5-α-cholestanol (R^2^ = 0.767, *P* < 0.0001). Each of the variables, when normalized by cholesterol, showed weaker correlations with DPI. The R^2^ was lowest for each of the non-cholesterol sterols alone. Repeated measures regression of plasma campesterol/5-α-cholestanol (Fig. 4A, *P* < 0.0001) and total phytosterols/5-α-cholestanol (Fig. 4B, *P* < 0.0001) on DPI were highly significant.

**Table 2:** Simple regression of outcome variables on dietary phytosterol intake.

Variables	R^2^
Non-cholesterol sterols normalized by cholestanol	
Plasma total phytosterols/5-α-cholestanol	0.767
Plasma campesterol/5-α-cholestanol	0.785
Plasma sitosterol/5-α-cholestanol	0.687
Plasma stigmasterol/5-α-cholestanol	0.551
Non-cholesterol sterols normalized by cholesterol	
Plasma total phytosterols/cholesterol	0.462
Plasma campesterol/cholesterol	0.512
Plasma sitosterol/cholesterol	0.395
Plasma stigmasterol/cholesterol	0.367
Non-cholesterol sterols alone	
Plasma total phytosterols	0.413
Plasma campesterol	0.444
Plasma sitosterol	0.317
Plasma stigmasterol	0.347

Results are R-squares of simple ordinary least square regression analysis (n = 94 observations), all *P* < 0.0001.

## Discussion

Dietary phytosterol intake cannot be quantified accurately in most studies because the phytosterol content of many individual foods is not available in major food databases. Therefore, we sought to identify a plasma biomarker for DPI. The principal finding is that the most suitable biomarker of DPI is the ratio of plasma campesterol to the cholesterol metabolite 5-α-cholestanol. The ratio of total plasma phytosterols to 5-α-cholestanol also was highly correlated with DPI.

Plasma phytosterol levels alone are not ideal biomarkers for DPI because circulating phytosterols reflect dietary intake, sterol absorption efficiency, and biliary excretion rate of absorbed phytosterols, the latter two of which comprise overall phytosterol handling by the body. Moreover, dietary phytosterols reduce the absorption efficiency of cholesterol, and probably the absorption efficiency of phytosterols themselves and other non-cholesterol sterols such as 5-α-cholestanol. At high levels of DPI, plasma phytosterol levels increase but this rise is blunted due to lower sterol absorption efficiency from a higher DPI or due to a lower intrinsic absorption capacity, which underestimates DPI. Conversely, higher sterol absorption efficiency, resulting from low DPI or a higher intrinsic absorption capacity, raises plasma phytosterol levels further, which overestimates DPI. Therefore, normalizing plasma phytosterols by a marker of non-cholesterol sterol metabolism is expected to improve the estimation of DPI. In our study, the coefficient of determination (R^2^) for DPI using ordinary least squares regression analysis increased when plasma campesterol and total phytosterols were normalized by plasma 5-α-cholestanol (Table 2).

It has been recognized that large variability exists in the response of serum cholesterol levels to dietary modification or drug therapy among individuals [18]. Similarly, large inter-individual variability exists in the handling of non-cholesterol sterols, as demonstrated by varied plasma campesterol concentrations at the same DPI level (expressed as mg phytosterols per kg of body weight) (Fig. 3). Interestingly, plasma 5-α-cholestanol levels also varied greatly at the same DPI level (Fig. 3). More importantly, plasma campesterol and 5-α-cholestanol were positively correlated at each phytosterol dose in both studies, suggesting that 5-α-cholestanol is handled similarly to phytosterols by lipid transporters. This same positive association also was found in the combined data in our studies and previously in free-living middle-aged men where DPI was not controlled [15]. Coordinate regulation of phytosterols and 5-α-cholestanol also has been reported in animals with ABCG5/8 deficiency, where both phytosterols and 5-α-cholestanol were increased in plasma and tissues, whereas cholesterol was reduced [6].

It is interesting to note that plasma 5-α-cholestanol was negatively associated with DPI in this study, consistent with a previous negative correlation between plasma 5-α-cholestanol and fecal phytosterols (the latter reflecting dietary phytosterol intake) in middle-aged men [15]. This strengthened the notion that plasma campesterol and 5-α-cholestanol are indeed coordinately regulated and this regulation occurred within a wide range of dietary phytosterol intakes. Plasma 5-α-cholestanol and phytosterols are regulated by similar factors in absorption and excretion except that 5α-cholestanol is synthesized by the body but phytosterols are not [15]. In addition, phytosterols appear to compete with the absorption of 5-α-cholestanol as suggested by a previous positive correlation between dietary phytosterols and fecal 5-α-cholestanol [15]. The diverging slopes between different DPI levels in Fig. 3 indicate that plasma 5-α-cholestanol level rose at a slower rate at a higher DPI. The differences in slope may be related to competition by phytosterols for absorption or excretion of 5-α-cholestanol, or alternatively to effects on the synthesis of 5-α-cholestanol.

Relatively little is known about the role of 5-α-cholestanol in the body [19]. 5-α-Cholestanol has been used as a marker for cholesterol absorption efficiency in healthy individuals, as well as in those with hyperlipidemia and type 2 diabetes [7,9,15,20,21]. In our study, however, the positive correlation of 5-α-cholestanol with % cholesterol absorption was relatively small by itself and when normalized by cholesterol (data not shown). Furthermore, there was no correlation between % cholesterol absorption and plasma 5-α-cholestanol when data were analyzed by DPI level (data not shown). Finally, the R^2^ was smaller (0.546) when plasma campesterol was normalized by the measured cholesterol absorption rather than plasma 5-α-cholestanol (0.785). Therefore, plasma 5-α-cholestanol appears to be more closely related to overall handing of phytosterols, rather than cholesterol, by the body.

The data from our controlled feeding studies enabled us to identify a plasma biomarker for DPI. These results need to be extended to a variety of diets that are typically consumed and in larger samples. For diets with different phytosterol compositions, the ratio of plasma total phytosterols to 5α-cholestanol might be preferred. Determination of non-cholesterol sterols must be performed by mass spectrometry and more work is needed to reduce costs and increase the standardization of these relatively new methods [22].

In addition to their robust effects on cholesterol metabolism, phytosterols may have many other benefits, such as anti-inflammatory and anti-cancer effects [4,5]. The plasma biomarker identified herein may prove to be useful in investigating the effects of dietary phytosterols on many biological, physiological, and pathological processes.
